# Towards actionable research frameworks for sustainable intensification in high-yielding rice systems

**DOI:** 10.1038/s41598-020-63251-w

**Published:** 2020-06-19

**Authors:** Meng-Chun Tseng, Alvaro Roel, Enrique Deambrosi, José A. Terra, Gonzalo Zorrilla, Sara Riccetto, Cameron M. Pittelkow

**Affiliations:** 1 Department of Crop Sciences, University of Illinois at Urbana-Champaign, Urbana, Illinois 61801 USA; 2 National Institute of Agricultural Research (INIA), Treinta y Tres, Uruguay; 3 Department of Plant Sciences, University of California, Davis, CA 95616 USA

**Keywords:** Agroecology, Environmental impact

## Abstract

New research frameworks that simultaneously address production and environmental goals are required to identify promising sustainable intensification options in high-yielding cereal systems. Here we estimated potential changes in environmental footprint associated with crop management practices aimed at breaking the yield ceiling for rice production in Uruguay. Results from a regional survey were combined with field experiments to integrate impacts on productivity and sustainability at two different intensification levels (average-yielding and high-yielding). Survey results indicate that high-yielding farmers produced 14% more grain compare to the regional average (7900 kg ha^−1^), with 25% to 99% lower agrochemical contamination risk and similar nitrogen use efficiency and carbon footprint. In on-farm trials, the alternative management practices increased yield beyond that of high-yielding farmers by up to 7% in small plots (8 site-years) and 15% in field-scale comparisons (6 site-years), yet an *ex post* assessment of environmental indicators shows significant decline of resource use efficiencies and increased carbon footprint. Thus, yield gains were not able to compensate for increased environmental footprint, highlighting the challenge of advancing the dual goals of SI in production systems nearing the yield ceiling. This study provides a simple but powerful framework for advancing SI in mainstream cereal production systems based on cost-effective modifications to existing agronomic experiments.

## Introduction

One of the greatest demands on world food systems is to increase production using fewer resources while at the same time reducing negative environmental consequences^1,2^. In response to such challenges, sustainable intensification (SI) has emerged as a set of guiding principles for simultaneously improving agricultural sustainability and productivity^3–5^. Compared to other approaches, SI is goal-oriented^6,7^, emphasizing the need to enhance food production with a reduced environmental footprint on existing farmland, rather than focusing on a specific means. Although SI was originally proposed within the context of smallholder farming systems in Africa^8^, it has gained global popularity because it integrates multiple disciplines, technologies, and locally-available resources to develop context-specific solutions.

Despite the concept of SI now being widely promoted by governments, local and international research institutions, and agribusiness^9^, most examples of SI implementation are limited to developing countries^10–12^. When yields are lower, the opportunity for increasing both productivity and sustainability is higher^13^. In contrast, the concept of further increasing yields while limiting environmental costs to achieve SI has been less investigated in high-yielding systems^14^. Although high levels of production contribute to global food security, recent estimates show that the majority of agriculture’s negative environmental consequences can be attributed to a few commodities produced in a few countries^15^. In addition to the need for reduced environmental impacts in these systems, future yield increases are uncertain because a significant proportion have experienced yield stagnation or plateaus in recent decades^16,17^. Lack of exploitable land is another constraint, as opportunities for extensification are generally lower and conversion of agricultural land into other uses is often associated with economic development^18^. Thus a key question for cereal systems nearing the yield ceiling is whether future production increases can be achieved without compromising environmental performance.

Although a number of studies have reported evidence of SI in high-yielding contexts^14,19,20^, these are primarily based on historical data and do not provide insights into management practices that farmers can adopt to increase future crop productivity. Concerns have also been raised that previous studies tend to emphasize yield over environmental goals^21^. When evaluating options for closing yield gaps, lower-yielding farmers are often compared with higher-yielding farmers^22,23^, which is helpful for identifying short-term opportunities to improve production, assuming average farmers can adopt high-yielding practices. But to achieve SI in systems nearing yield potential, it is also necessary to enhance yields and environmental performance of top-yielding farmers. However, little research has occurred in this area. While participatory research approaches have been widely used to forge more sustainable production in developing countries^24,25^, we are unaware of efforts to engage top-yielding farmers and explicitly use their management practices as a baseline for improving crop productivity in high-yielding systems. To better leverage available resources and accelerate progress towards SI, agriculture is in need of cost-effective, novel research frameworks that simultaneously address production and environmental goals.

Rice is one of the most important staple food crops in the world, representing around 50% of total dietary caloric intake^26^. Rice production is also associated with important environmental concerns. Increased fertilizer nitrogen (N) application has been a main approach for boosting yields in previous decades, but this has resulted in low N recovery and widespread pollution^27,28^. Large fossil fuel and embodied energy inputs can decrease net energy yield and energy use efficiency^29,30^. When energy inputs are converted to CO_2_ equivalents, intensive production practices are associated with a high carbon footprint (CF), adding to the significant field greenhouse (GHG) emissions occurring in rice compared to other cereal crops^31^. Agrochemical inputs such as herbicides, pesticides, and fungicides also increase environmental footprint, requiring resources to produce and potentially having negative impacts on surrounding ecosystem^32,33^. Given the importance of rice in meeting global food security goals, comprehensive studies based on a range of resource use efficiency and environmental indicators are required to identify benefits and drawbacks of management strategies in different production contexts^34,35^.

Rice has been cultivated in Uruguay in rotation with pastures since the early 1930s and occupies around 160,000 ha annually. At present, over 95% of production is exported to the global market, making Uruguay the 7^th^ largest rice exporter in the world^36^. In order to remain economically competitive, the rice sector is uniquely structured to produce high quality long grain rice to meet consumer demand through a tightly integrated value chain linking research, production, and exports industries. For further background see Blanco *et al*. and Zorrilla *et al*.^37,38^. In recent decades, technological changes in agronomic management have increased average farm-level productivity by 60–70% (from 5 to 8.5 Mg ha^−1^)^39^, leading to increased net energy yield and water use efficiency and decreased carbon footprint per unit of production^19^. Average yields are now the 3^rd^ highest in the world, despite the absence of government subsidies that farmers often receive in other countries, and important progress has been made in balancing crop production and agricultural sustainability outcomes. However, similar to other high-yielding cereal systems^13,16^, there are serious concerns about yield stagnation and new approaches are needed to further boost productivity, particularly for top-yielding farmers.

In this study, we explored strategies for SI within the context of Uruguay rice production by combining crop production measures with an evaluation of sustainability indicators at two different scales: (1) a comparison of high-yielding farmers against the average based on survey information and national production statistics, and (2) a series of on-farm field experiments designed to increase yield for high-yielding farmers. Our specific objectives were to evaluate if potential yield increases could be achieved with a reduced environmental footprint compared to baseline management practices at these two different intensification levels, helping address the question of whether trying to increase the yields of average-yielding vs. high-yielding farmers holds greater potential for making progress towards SI.

## Methods

### Comparison between average-yielding and high-yielding farmers

**Figure 1:**
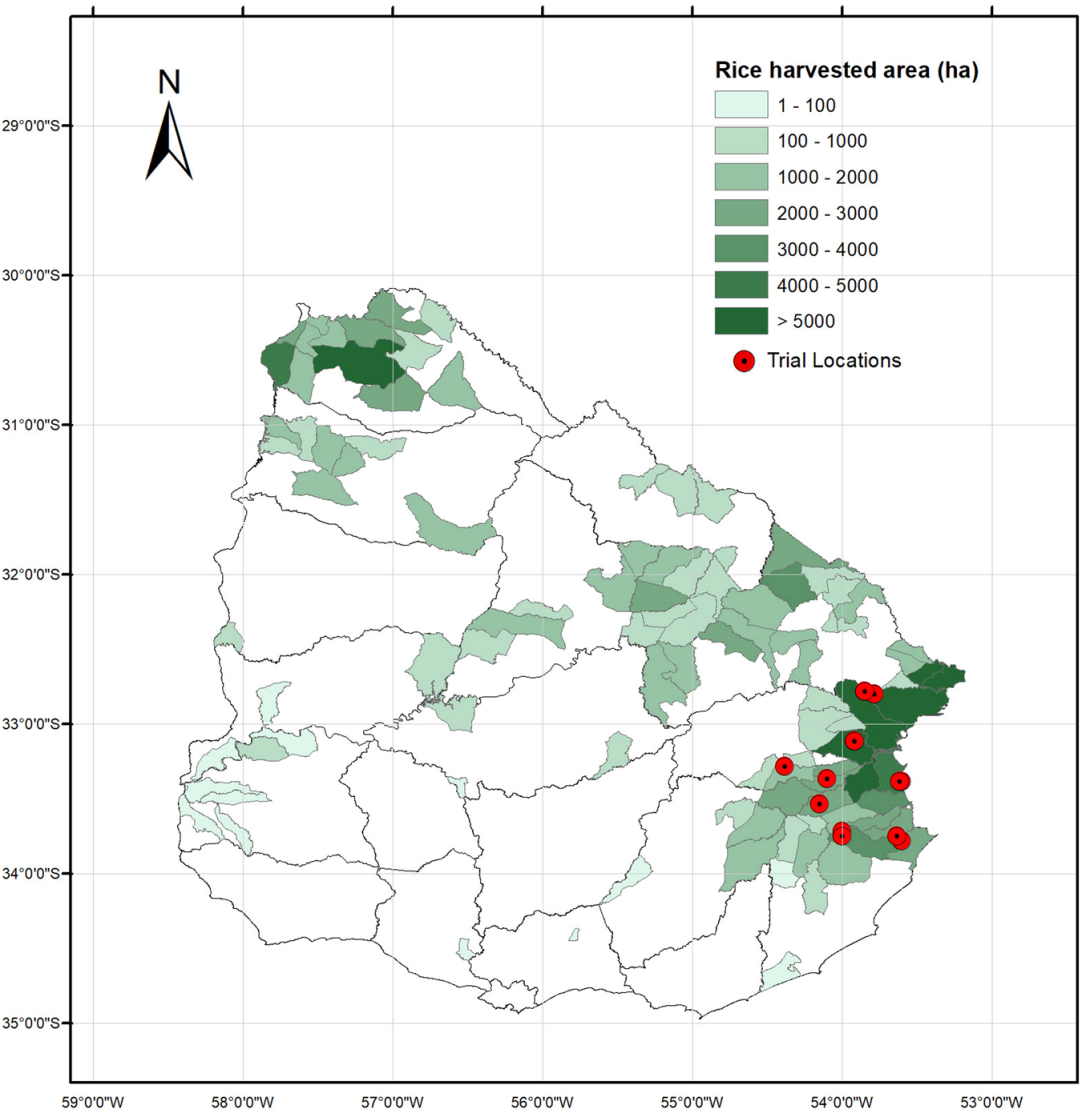
Map of rice production in Uruguay. Red dot indicates the sites of on-farm trails discussed in this study. Source: Created by Carracelas G., INIA, Uruguay.

Farm surveys were carried out jointly by scientists of National Institute of Agricultural Research of Uruguay (Spanish acronym: INIA) and the three major commercial rice mills in Uruguay (Saman, Coopar and Casarone) to identify current management practices associated with high productivity in the eastern rice growing region of Uruguay. Eastern Uruguay accounts for approximately 70% of national production area each year (Fig. 1). In this study, high-yielding farmers were defined as those having yields greater than top 20% quantile over three consecutive seasons (2011–2014). Questionnaires were administered to obtain crop management information from this subset of farmers including soil type, irrigation system, tillage practices, cultivar, seeding rate, fertilizer application type and rate, and pesticide application, if applicable, among other variables. As explained in Pittelkow *et al*.^19^, management records are customarily shared between farmers and millers in Uruguay to develop agronomic recommendations. In total, survey information was collected from 39 farmers covering the three main rice production sub-regions within eastern Uruguay (15 in Treinta y Tres, 14 in Cebollatí, and 10 in India Muerta) (Supplementary Table S1). This information was then synthesized to develop 20 consensus management practices in each production sub-region based a joint meeting with representatives for INIA, the Rice Growers Association (Spanish acronym: ACA), the Union of Commercial Rice Millers (Spanish acronym: GMA) and Coopar (a commercial rice miller) (Supplementary Table S2).

The regional average of yield in eastern Uruguay was used as a reference for comparison. National records on rice cultivation from 3 seasons (2011–2014) were used to estimate yield, resource use efficiency, and environmental footprint in the three main production regions. The methods for calculating indicators followed Pittelkow *et al*.^19^, except that water use efficiency was excluded due to lack of field-specific irrigation records for high-yielding farmers and area-specific records for the regional average. We are aware that the current indicators cannot fully represent the broad spectrum of agricultural sustainability. In addition to the need for more environmental indicators, a more holistic approach to SI would include social and economic dimensions. For context, the reader is referred to other recent studies which provide a comprehensive review of indicators and SI research^40–42^.

### On-farm experiments to break the yield ceiling

On-farm field experiments were conducted in two stages to test the possibility of further enhancing productivity in this region using high-yielding farmer management practices as a reference point. The first stage trials evaluated a wide range of management options for maximizing yield in different sub-regions, whereas the second stage trials validated the performance of the highest yielding treatment at the farm-scale based on the results from the first stage trials. All trials are conducted in the Eastern rice production region (Fig. 1). In the first stage, 12 treatments were evaluated at 4 sites over two seasons (season 2014–2015 and 2015–2016). In the second stage, the management practice for each sub-region resulting in the greatest yield improvement was further evaluated at 6 sites in one season (season 2016–2017). All trials were located in farmers’ fields who were identified previously as high-yielding farmers, with each farmer performing field operations and management practices with guidance and supervision from INIA.

Sites for the first stage trials were located near Rincon de Ramirez (32°47′ S 53°51′W for 1^st^ season; 32°48′S 53°47′W for 2^nd^ season), Treinta y Tres (33°22′S 54°06′W for 1^st^ season; 33°07′S 53°55′W for 2^nd^ season), Cebollati (33°23′S 53°36′W for 1^st^ season; 33°23′S 53°37′W for 2^nd^ season), and India Muerta (33°43′ S 54°00′W for 1^st^ season; 33°47′S 53°36′W for 2^nd^ season), respectively (for the detailed locations on the map, readers can refer Supplementary Fig. S1a in Supplementary Materials). The baseline treatment, or control in this experiment, was designed following the 20 consensus management practices for each sub-region described above (hereafter labeled high-yielding farmer practice (HYFP)). To break the yield ceiling, a joint meeting between INIA, extension specialists from the commercial rice mills, and high-yielding farmer representatives was held in early 2014 to develop an integrated management package that had the potential to further increase yields based on research, extension, and farmer experience with alterations to individual management components. This treatment (hereafter labeled best management practice package, BMPP) was composed of five management modifications (Table 1): (1) Improved cultivars with greater yield potential and disease resistant, (2) Seeding rate based on desired plant density and the use of alternative seed treatment, (3) Soil-test based fertilization rate for nitrogen (N), phosphorus (P), and potassium (K), (4) Application of sulphur and silica micronutrients, and (5) Modified disease protection methods. As this study reports on the broader implications on the research framework but not the specific outcomes of agronomic management changes, the detailed information on farm-level operations are shown under Supplementary table S3.

**Table 1:** Description of management practices for high-yielding farmer practice (HYFP) and best management package proposed (BMPP) employed in on-farm trials.

	Feature	Management package	Description
1	Cultivar	HYFP	Common cultivars adopted by local farmers.
		BMPP	Newly released cultivars with increased yield potential and rice blast resistant.
2	Seed and seed treatment	HYFP	Seedling with fixed rates (varies by location); Seed were treated with fungicide and insecticide.
		BMPP	Seedling rates were determined by seed size, germination rate and field survival rate with the goal of 180 plants per square meter. Seeds were treated with identical pesticide as HYFP, with additional commercialized treatment of zinc and nitrogen-fixing bacteria *Herbaspirillum*.
3	Fertilization rate	HYFP	N, P and K rates were determined by farmers.
		BMPP	N, P and K rates were determined by the soil test results before land preparation. Fertilizer rate was designed with anticipated crop removal at 12 Mg ha^−1^ grain yield.
4	Micronutrient application	HYFP	No additional micronutrients were applied.
		BMPP	Sulfur, silicon and commercial micronutrients products were applied.
5	Disease management	HYFP	Fungicide is treated during panicle initiation stage. Second fungicide and insecticide are applied only when necessary.
		BMPP	Fungicide is treated during panicle initiation stage. Second fungicide application is replaced by beneficial agent.

To compare the complete BMPP against HYFP and also examine the contribution of individual components, 12 treatments were implemented following the omission plot design^43^. With this approach, the first set of treatments includes components added individually to the control (HYFP), while the second set of treatments includes components subtracted individually from BMPP. A true factorial design was not possible due to the large number of individual components resulting in an unrealistic amount of treatment combinations. Thus, at each site the following 12 treatments were implemented in a randomized complete block design (RCBD) with 3 replications. Treatment 1 was HYFP while treatment 7 was BMPP. Treatments 2 to 6 consisted of HYFP with one component replaced by its corresponding BMPP practice (e.g. addition of improved cultivar). Likewise, treatments 8 to 12 consisted of BMPP with one component replaced by its corresponding HYFP practice (e.g. use of original instead of improved cultivar). Plot sizes were 122.4 m^2^. Management practices and input information were recorded including tillage method, fertilizer rate, and agrochemical application for each treatment and location. Yield of each plot was recorded based on cleaned and dried rice grain with a moisture content of 13%.

Sites for the second stage trials were located near Rincon de Ramirez (32°47′S 53°51′W), San Francisco (33°07′S 53°55′W), Treinta y Tres (33°17′S 54°23′W), Cebollati (33°45′S 53°38′W), India Muerta (33°45′S 54°00′W) and Vuelta Grande (33°32′S 54°09′W) (Supplementary Fig. S1b). These were larger, side-by-side trials where a field approximately 10 ha in size was divided equally between HYFP and the second stage BMPP. The second stage BMPP for each location was selected based on the treatment resulting in the highest yield increase in the first stage trials. This decision was made in each sub-region jointly by INIA researchers and farmers with the main goal to achieve the highest yield. As in stage one trials, treatment details for HYFP differed from location to location since the consensus set of high-yielding management practices in each location was adapted to local environmental conditions. Treatment 9 (BMPP without the seed treatment method) was selected for five trials located near Rincon de Ramirez, San Francisco, Treinta y Tres, India Muerta and Vuelta Grande. Treatment 12 (BMPP without the advanced disease protection method) was selected for one trial near Cebollati. Other trial management and harvest activities followed that of the first stage trials. The detailed information for farm-level operations in this trial are reported under Supplementary table S4.

### Evaluation of environmental sustainability indicators

To understand possible synergies or tradeoffs between yield and environmental sustainability for the different treatments, inputs associated with each treatment were used to calculate resource use efficiency and environmental impact indicators following Pittelkow *et al*.^19^, with several slight modifications. For instance, water input and water productivity calculations were not conducted due to lack of on-site irrigation information. Resource use efficiency metrics covered nitrogen use efficiency (NUE), energy use efficiency (EUE), and net energy yield (NEY). Environmental impacts were represented by carbon footprint (CF) and agrochemical contamination risk (ACR).

Nitrogen and energy use efficiency were calculated as partial factor productivity of N and energy, respectively (i.e. grain yield divided by either total N input or energy input). Energy inputs in this study were calculated using only on-farm input data (i.e. fertilizer rate, seed rate, diesel usage and agrochemical rate), while other factors such as labor input, diesel and electricity for irrigation, and processing and transportation of harvested grain were not considered due to lack of data. Hence, energy efficiency focused on management-induced changes related to the treatments in this study, rather than quantifying total energy use of the system. Importantly, if practices such as irrigation and grain handling were included, these would be relatively consistent across treatments. Net energy yield represented the total embodied energy of harvested grain minus the energy inputs. CF was calculated based on the amount of diesel used in field activities (land preparation, application of seed, fertilizer, and pesticides, harvest), and embodied energy contained in seed, fertilizer, and agrochemical inputs. Similar to energy efficiency, CF was focused on management-induced changes and was only partial as it excluded energy required for irrigation and field emissions of nitrous oxide and methane. For better representation of carbon footprint impacts on production, yield-scaled carbon footprint (YSCF) was calculated by dividing CF values by yield. Detailed background information regarding the embodied energy of inputs and conversion factors were described in the Supplementary Materials of Pittelkow *et al* .^19^.

Agrochemical contamination risk (ACR) to freshwater ecosystems was calculated using the USEtox database^33^. This indicator reflects the potential impact on aquatic species per volume (m^3^) of fresh water, determined as the product of ecotoxicity effect factor and the amount of active ingredient for each agrochemical. The calculations is based on the summarized management practice of farmer survey and reported pesticides usage in seed treatment and field-level disease control in on-farm trails (refer Supplementary File S1 raw data of summarizing USEtox ecotoxicity effects of individual treatments). Yield-scaled ACR (YSACR) was calculated by summing the impacts of individual agrichemicals and dividing by yield.

### Statistical analysis

Analysis of variance (ANOVA) was performed using SAS PROC Mixed version 9.4. The model for the first stage trials followed a randomized complete block design with location nested within year, block nested within location and year, and the corresponding interactions of treatment with year and location:

$${X}_{ijkl}=\mu +{Y}_{i}+{L}_{(i)j}+{B}_{(ij)k}+{\tau }_{l}+Y{\tau }_{il}+L{\tau }_{(i)jl}+{\varepsilon }_{ijkl}$$

where,

*X*_*ijk*_ represents the observation value under i^th^ year, j^th^ location, k^th^ replication and l^th^ treatment

*μ* is the grand population mean

Y_i_ is the random effect associated with i^th^ year

*L*_(*i*)*j*_ is the random effect associated with j^th^ location, nested by i^th^ year

*B*_(*ij*)*k*_ is the random effects of associated with k^th^ block, nested by i^th^ year and j^th^ location

*τ*_*l*_ is the fixed effect associated with l^th^ treatment

*Yτ*_*il*_ is the interaction between year and treatment

*Lτ*_(*i*)*jl*_ is the interaction between location and treatment

*ε*_*ijkl*_ is the error associated with individual observation

The model of the second stage trial accounted for variance associated with both location and treatment:

$${Y}_{ij}=\mu +{\tau }_{i}+{L}_{j}+{\varepsilon }_{ij}$$

where

*Y*_*ij*_ is the observation of i^th^ treatment and j^th^ location

*μ* is the grand population mean

*τ*_*i*_ is the fixed treatment effect associated with i^th^ treatment

*L*_*j*_ is the random location effect associated with j^th^ location

*ε*_*ij*_ is the random error associated with i^th^ treatment in j^th^ location

The assumption of normality was examined using Shapiro-Wilk test in proc univariate of SAS. The assumption of homogeneity of variance was validated using Brown-Forsythe modification of the Levene test in the MEANS option of PROC GLM. To compare treatment effects, Fischer’s protected LSD was used as a post-hoc test (*α* = 0.05).

## Results

### Comparison of yield and environmental sustainability indicators between average and high-yielding rice farmers

At the average-yielding intensification level, yield and sustainability indicators were compared for high-yielding farmers to the regional average using survey data from 2011–2013. Overall, yield increases were generally accompanied with a reduced environmental footprint in terms of agrichemical contamination risk and carbon footprint. Yields for the high-yielding farmers ranged from 8,986 to 9,025 kg ha^−1^ in the surveyed sub-regions (Treinta y Tres, Cebollati and India Muerta), which was around 14% higher than average yield in eastern Uruguay (Table 2). Values for NUE and net energy yield NEY of high-yielding farmers were 5.1 to 5.5% and 13.8 to 16.4% higher than the regional average, respectively. There were several regional differences in EUE, with high-yielding farmers having 2% lower EUE in India Muerta but 16.4% and 6.8% greater EUE in Trienta y Tres and Cebollati, respectively. ACR was reduced by 97.7%, 98.5% and 24.3% and YSACR reduced by 98%, 99% and 33.2% in Trienta y Tres, Cebollati and India Muerta, respectively. Total CF slightly increased (1.3% to 2.8%) for high-yielding farmers compared to the average, whereas YSCF decreased by 10.3% to 11.5%.

**Table 2:** Yield and environmental performance of high-yielding farmers in the three main production sub-regions of eastern Uruguay compared to the regional average. ^*^The values within parentheses represent the relative change compared to the regional average in percentage.

Variables	Regional average	High-yielding farmers		
		Trienta y Tres	Cebollati	India Muerta
Yield (kg ha ^−1^)	7900	8986 (13.7%)^*^	9025 (14.2%)	9025 (14.2%)
NUE (kg yield kg applied N^−1^)	121.68	128.37 (5.5%)	127.83 (5.1%)	127.83 (5.1%)
Net energy yield (GJ ha ^−1^)	102.94	119.84 (16.4%)	118.86 (15.5%)	117.20 (13.8%)
Energy Use Efficiency (kg yield MJ^−1^)	0.4609	0.5366 (16.4%)	0.4927 (6.8%)	0.4519 (−2%)
Agrochemical contamination risk (PAF m^3^)	15608.75	352.82 (−97.7%)	236.88 (−98.5%)	11815.19 (−24.3%)
Yield-scaled Agrochemical contamination risk (PAF m^3^ kg yield^−1^)	1.96	0.04 (−98%)	0.02 (−99%)	1.31 (−33.2%)
Carbon Footprint (kg CO_2_ eq. ha^−1^)	7524.17	7636.49 (1.5%)	7621.2 (1.3%)	7731.33 (2.8%)
Yield-scaled C footprint kg (CO_2_ eq. kg yield^−1^)	954.75	849.82 (−11%)	844.45 (−11.5%)	856.70 (−10.3%)

### Evaluating opportunities to improve yield and sustainability for high-yielding farmers

At the high-yielding intensification level, different strategies for SI were tested using small-plot trials in the first stage and large-plot trials in the second stage combined with an ex-post sustainability evaluation. The first stage trials included a wide range of management options for maximizing yield in different sub-regions, whereas the second stage trials validated the performance of the highest yielding treatment at the farm-scale based on the results from the first stage trials. Yield increases and potential tradeoffs associated with yield increases were both evaluated. Overall, results indicate that while yield increases are achievable, environmental tradeoffs are more likely compared to the lower baseline yield level of average farmers. Aggregated results across all regions and years of individual indicators in the field trials are presented in the following paragraphs. In this paper, we aimed to provide general conclusions of the experiments and therefore did not present results at regional level. For regional-specific responses, readers are referred to Supplementary Table S5.

**Figure 2:**
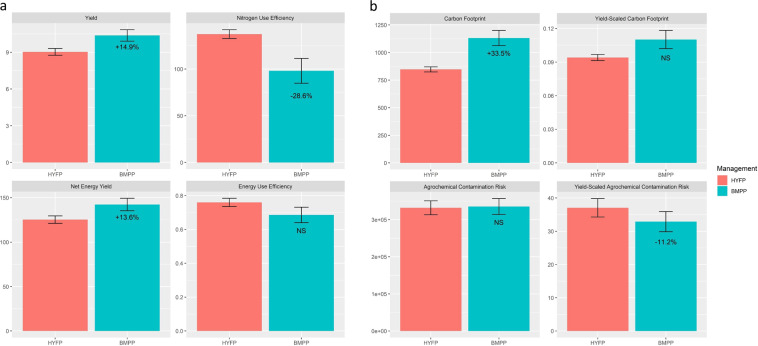
Performance measurements of (**a**) yield and resource use efficiency (**b**) environmental footprint of the second stage trials average across 6 site-years of second-stage trial. Values represent the relative change compared to HYFP. The range of error bar represents 2 standard error of the mean. Values displayed under the error bars represent the percentage change relative to HYFP. For the unit of each indicator, refer Table 4. NS: not significant.

#### Analysis of variance

In the first stage trials, the effects of treatment, location, and location by treatment interactions were significant for all 6 indicators (Table 3). P-values for location (nested within year) and location by treatment interactions were highly significant, while p-values for treatments were all greater than 0.001 (except NUE and YSACR). No significant difference between the two years was found for all 6 indicators. In the second stage trials, significant differences between treatments were detected for yield, NUE, NEY, CF and YSACR, but not for EUE, YSCF and ACR (Table 4). A significant location effect was detected only for ACR and YSACR.

**Table 3:** Analysis of variance results for the first stage field trials conducted across four sites and two years.

Factors	P-value			
	Location (Year)	Treatment	Year	Location × Treatment
Yield	<0.0001	0.0015	NS	<0.0001
Nitrogen Use Efficiency	<0.0001	<0.0001	NS	<0.0001
Net Energy Yield	<0.0001	0.0155	NS	<0.0001
Energy Use Efficiency	<0.0001	0.0099	NS	<0.0001
Yield Scaled C Footprint	<0.0001	0.0111	NS	<0.0001
Yield Scaled AGC Contamination	<0.0001	<0.0001	NS	<0.0001

**Table 4:** Yield and sustainability performance for the second stage trials average across 6 site-years. Values in bold represent a significant difference between HYFP and BMPP. NS: not significant.

Variables	Mean			P-value	
	HYFP	BMPP (proposed)	Change (%)	Treatment	Location
**Yield and resource use efficiency**					
Yield (Mg ha ^−1^)	9.03	10.37	**14.9%**	0.01	NS
Nitrogen Use Efficiency (kg yield kg applied N^−1^)	137.35	98.12	− **28.6%**	0.04	NS
Net Energy Yield (GJ ha ^−1^)	125.35	142.36	**13.6%**	0.03	NS
Energy Use Efficiency (kg yield MJ^−1^)	0.76	0.69	−9.8%	NS	NS
**Environmental footprint**					
Carbon Footprint (kg CO_2_ eq. ha^−1^)	847.25	1131.17	**33.5%**	0.02	NS
Yield-scaled Carbon Footprint (kg CO_2_eq kg yield^−1^)	0.094	0.110	17.0%	NS	NS
Agrochemical Contamination Risk (PAF m^3^)	331925	335481	1.1%	NS	<0.01
Yield-scaled Agrochemical Contamination Risk (PAF m^3^ kg yield^−1^)	37.06	32.9	− **11.2%**	0.02	<0.01

#### Treatment effects on yield

Grain yield varied significantly in response to the different management packages in the first stage trials (Tables 3 and 5). Combining results of 2 seasons and 4 locations, BMPP (treatment 7) was 4.2% higher than HYFP. Yields in treatments 2–6 (where single components of BMPP were added to HYFP) were not significantly different from HYFP (treatment 1), with yield differences ranging from −1% to 1.5%. Yields in treatments 8–12 (where single components were subtracted from BMPP), were not significantly different from BMPP (treatment 7), except for treatment 8 (without improved cultivar). However, when comparing treatments 8–12 to treatment 1 (HYFP), several of these showed a significant yield increase (5.4% to 6.9%), slightly higher than plain BMPP (4.2%), indicating that management packages combining elements of both HYFP and BMPP approaches might out-yield BMPP.

**Table 5:** Yield and sustainability performance for the first stage field trials averaged across 8 site-years.

	Treatment	Yield		NUE		NEY		EUE		YSCF		YSACR	
		Mg ha^−1^		kg yield kg applied N^−1^		GJ ha ^−1^		kg yield MJ^−1^		kg CO_2_eq kg yield^−1^		PAF m ^3^ kg yield ^−1^	
1	HYFP	11.62		167.55		165.27		0.973		0.075		29.75	
2	+Improved Cultivar	1.5% ^##^		1.5%		2.3%		2.2%		−1.8%		−1.8%	
3	+Seed Technology	−0.8%		−0.8%		−0.1%		4.0%		−3.0%		**−21.8%**	
4	+Fertilization	0.9%		**−18.9%**		0.1%		**−10.4%**		**15.3%**		−1.3%	
5	+Micronutrient	−0.3%		−1.2%		0.4%		0.0%		0.1%		−0.7%	
6	+Plant Protection	−1.0%		−1.2%		−1.4%		−1.2%		1.0%		1.1%	
7	BMPP	12.10	4.2%^*^	147.59	− **11.9%**	171.06	3.5%	0.93	−3.8%	0.081	**8.1%**	25.36	**−14.8%**
8	−Improved Cultivar	− **4.3%**	−0.4%	−3.9%	− **15.4%**	−3.7%	−0.3%	−1.3%	− **5.1%**	1.9%	**10.2%**	− **8.9%**	− **22.3%**
9	−Seed Technology	2.3%	**6.6%**	2.1%	**−10.1%**	2.7%	**6.2%**	0.6%	−3.3%	−1.3%	6.7%	**9.6%**	**−6.6%**
10	−Fertilization	−2.0%	2.1%	**14.6%**	0.9%	−0.6%	2.9%	**8.4%**	4.2%	− **10.0%**	−2.7%	0.2%	− **14.6%**
11	−Micronutrient	1.2%	**5.4%**	−4.3%	**−15.7%**	1.6%	**5.1%**	−0.6%	−4.5%	0.3%	**8.4%**	−3.1%	**−17.4%**
12	−Plant Protection	2.7%	**6.9%**	3.1%	**−9.2%**	2.7%	**6.3%**	2.6%	−1.3%	−2.9%	5.0%	−4.1%	**−18.3%**

Treatment 1 is HYFP while treatment 7 is BMPP. Treatments 2 to 6 consist of HYFP with one component replaced by its corresponding BMPP practice, as indicated. Likewise, treatments 8 to 12 consist of BMPP with one component replaced by its corresponding HYFP practice, as indicated.

^#^The percentage value indicate the relative change compare to corresponding management package (HYFP or BMPP). ^*^The additional column indicates relative change compared to HYFP. ^*^Value in bold represents significant difference compare to corresponding complete management packages at 0.05 level under Fisher’s least significant difference (LSD).

In the second stage trials, treatment 12 was employed in Cebollati and treatment 9 in the remaining 5 locations as the second stage BMPP. Across all 6 sites, an average of 14.9% (p-value 0.01) (Table 4; Fig. 2a) yield increase was found. There was no significant location effect on yield. The yield increase with the second stage BMPP was approximately two times higher (14.9% vs 5.4–6.9%) than in the first stage trials (Table 4; Fig. 2a).

**Figure 3:**
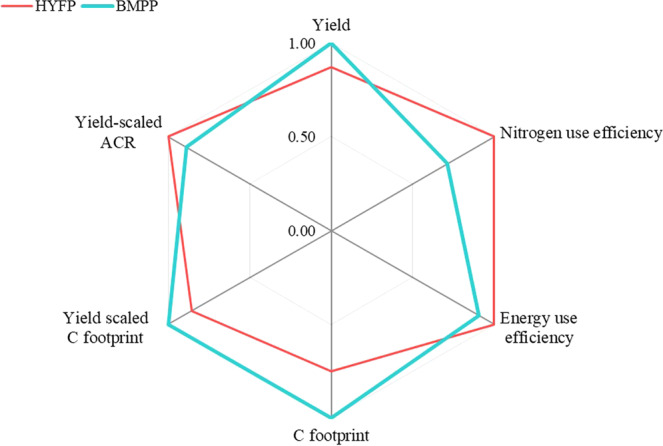
Comparison of 6 yield and environmental sustainability indicators between HYFP and BMPP at stage two field trials. The highest value of each indicator was normalized to 1.

#### Treatment effects on energy and resource use efficiency

Nitrogen use efficiency (NUE) varied significantly in response to the different management packages in the first stage trials (Tables 3 and 5). NUE in BMPP (Treatment 7) was significantly reduced by 11.9% compare to HYFP (Treatment 1). Within treatments 2–6, adopting the fertilization method from BMPP (treatment 4) reduced NUE by 18.9% compared to HYFP (treatment 1). In treatments 8–12, NUE was significantly reduced compared to HYFP, except in treatment 10 (BMPP without field test-based fertilization method). That is, under treatments 8–12, using fertilization rate from HYFP (Treatment 10) increased NUE by 14.6%, resulting in no significant difference compared to HYFP. In the second stage trials, BMPP on average decreased NUE by 28.6% across the 6 locations (Table 5).

Net energy yield (NEY) followed a similar pattern of changes as yield. Positive NEY was observed across all treatments, indicating greater return in embodied grain energy than energy investment in terms of seed, material, and fuel inputs (Table 5). In the first stage trials, no significant difference was found between HYFP and BMPP, or when treatments 2–6 were compared to HYFP and treatments 8–12 were compared to BMPP. However, treatments 9, 11, and 12 significantly increased NEY by 5.1–6.3% compared to HYFP, due to increases in yield. In the second stage trials, BMPP significantly increased NEY by 13.6% (Table 4; Fig. 2a).

Energy use efficiency (EUE) was significantly affected by treatment and location in the first stage trials (Table 3). Changes in EUE were largely in accordance with NUE (Table 5), however, in contrast to NUE no significant differences were found between HYFP and BMPP. Similar to NUE, the fertilization method is the main influential factor to EUE. For example, when the fertilization method of HYFP was replaced by the fertilization method of BMPP (i.e. treatment 4), there was a 10.4% reduction in EUE compared to HYFP. Conversely, when the fertilization method of BMPP was replaced by HYFP (i.e. treatment 10), there was an 8.4% increase in EUE compared to BMPP. No significant differences were found in response to the management treatments across the different locations in the second stage trials (Table 4).

#### Treatment effects on environmental footprint per unit of production

Yield-scaled carbon footprint (YSCF) was significantly affected by treatment in the first stage trials (Table 3), with an 8.1% increase in BMPP compared to HYFP (Table 5). When subcomponents of BMPP were subtracted individually (treatments 8–12), YSCF increased between 5 to 10% compared to HYFP, except for treatment 10. Similar to EUE, switching the fertilization strategy from HYFP to BMPP, or vice versa, resulted in a 15.3% increase, or 10% decrease, in YSCF, respectively. There was no change between HYFP and BMPP in YSCF in the second stage trials (Table 4; Fig. 2b).

Treatment significantly influenced YSACR in both the first (Table 3) and second stage trials (Table 4). In the first stage trials, BMPP reduced YASCR by 14.8% compared to HYFP (Table 4). Similarly, all BMPP treatments with single components removed (treatments 8–12) had significantly lower YSACR compared to HYFP. By adding the BMPP seed technology to HYFP (treatment 2), YSACR was reduced by 21.8% compared to HYFP, while removing the BMPP seed technology from BMPP increased YSACR by 9.6% compared to BMPP. In second stage trials, BMPP significantly reduced YSACR by 11.2% (Table 4; Fig. 2b).

## Discussion

### Opportunities towards sustainable intensification in uruguay rice systems

Considering that farmers tend to prioritize yield and profit over other outcomes^44^, we focused on identifying opportunities for yield gains as an initial entry point for SI practices while also accounting for potential environmental sustainability tradeoffs. The current context for rice production in Uruguay, as in many other countries, is that yield increases are one of the most important goals of research and extension activities^45^, thus our approach capitalized on available infrastructure and resources to identify management practices that could be easily adopted by farmers to enhance short-term sustainability outcomes. As discussed below, this marks an important distinction compared to other SI studies seeking more transformational changes in agriculture.

Based on the regional survey data for 2011–2013, we found that high-yielding farmers had higher NUE and EUE compared to the average, leading to greater net energy yield and reductions in carbon and agrochemical footprint per kg of rice yield (Table 2). This is a notable result, indicating that simultaneously achieving higher yield with increased resource use efficiencies and lower environmental impacts might be possible for average farmers in this region, assuming it is possible to optimize their management to reach yield levels similar to high-yielding farmers. We note this is a large assumption that must be explored further, yet it is promising that previous work has shown that improved crop management practices are a key factor contributing to higher on-farm rice yields in this system^46^. The finding that improved agronomy has led to yield increases with a reduction in environmental costs per unit production is also consistent with a national assessment of rice systems over the previous two decades in Uruguay^19^. Similar trends have been documented in other field experiments^47,48^ and on-farm trials^45^ for rice production in China, as well as for commodity crops including rice and maize the U.S.^20^. Even in cases where input levels have increased, higher yields can compensate leading to greater input use efficiencies^49^ or reduced carbon footprint^50^. Collectively these results provide further evidence that well-managed, intensified crop production systems can contribute to the dual goals of SI.

Although it is inherently difficult to conduct an SI transformation without the involvement of all major stakeholders^51^, our study suggests an important starting point is collaborative research with farmers. Kanter *et al*.^52^ previously described a transdisciplinary approach for casting different SI pathways for the Uruguayan beef sector, where environmental goals were established based on the performance of elite farmers and future pathways for increasing both yield and sustainability were explored by assuming that average farmers adopted the management practices of elite farmers. While future insights can be gained by benchmarking the performance of high-yielding farmers, once the yield ceiling has been (theoretically) approached by average farmers, new crop management strategies must in turn be developed to support continual yield increases. From an SI perspective, an unanswered question is whether the environmental implications of such practices have positive or negative impacts on sustainability. We uniquely addressed this question by conducting a set of follow-up participatory research experiments where high-yielding farmers were involved in the design and management of field trials with the aim of breaking the yield ceiling.

In the first stage trials, the individual components of BMPP did not significantly increase yield compared to HYFP (Table 5, treatment 2–6). However, the full BMPP increased yield by 4.2%, indicating the importance of evaluating management options holistically. As demonstrated in previous studies using this experimental design^43^, evaluating crop management packages instead of individual practices can help identify the synergistic effects of individual management options, particularly under intensively managed systems. Similar conclusions have been reached when evaluating other crop management packages that may contribute to synergistic effects, for instance the system of rice intensification (SRI) in India^53^. However, this does not suggest that all components contributed equally. Based on our results, using an improved cultivar might be the most crucial component of BMPP, as removal of the improved cultivar resulted in 4.3% yield decrease compare to full BMPP (treatment 8–12 vs. treatment 7), while removing other components did not significantly decrease yield.

When accounting for environmental performance, a significant finding is that no treatment simultaneously improved yield and the full suite of sustainability indicators compared to HYFP in either the first or second stage trials (Table 5; Fig. 3). Despite the yield increase observed with BMPP, there are limitations to this package due to environmental tradeoffs that occurred when the yield goal was prioritized. Specifically, under BMPP both NUE and EUE decreased and YSCF increased in both first and second stage trials, indicating that increased yield was not able to compensate for the additional environmental footprint relative to high-yielding farmers. Improved management of N fertilizer is critical for enhancing the sustainability of global food production^54^, thus decreasing NUE under BMPP must be weighed as a negative consequence against potential yield gains. However, it should be noted that current NUE levels for Uruguay are still high compared to other rice systems internationally regardless of elevated trend of N input in recent years^19^. For example, recent on-farm trials deploying best management practices in China suggested attainable NUE in single rice cropping systems around 56 kg rice kg^−1^ N, with a highest observed NUE of 80 kg rice kg^−1^ N^55^. Another study reported NUE ranging from 37 to 62 kg rice kg^−1^ N^56^. In comparison, the current BMPP practice in this study averaged 148 kg rice kg^−1^ N in the first stage trials and 98 kg rice kg^−1^ N in the second stage trials.

Another option for SI would be practices that decrease the environmental footprint of high-yielding farmer practices without a corresponding yield penalty. We found limited opportunities for this, the main reason being that the BMPP treatments required greater N inputs than HYFP. On the basis of soil test results, higher N application rates were applied for BMPP in 7 out of 8 site-years compared to HYFP (Table S3). Increased carbon footprint and decreased energy efficiency resulted from increased inputs of both N and energy, indicating that future optimization of BMPP practices to achieve the observed yield increases without increasing N inputs might be a possible SI solution. As rice is commonly rotated with perennial pastures in Uruguay, adopting legume-based improved pastures as suggested by Kanter *et al*.^51^ could serve as a biological source of N in this system, reducing the need for external N fertilizer while still supporting yield increases in the future.

The multi-criteria approach in this study allowed us to quantify benefits and tradeoffs of potential yield increases at different levels of intensification (i.e. average-yielding vs. high-yielding farmers). For example, most indicators would theoretically be improved if average-yielding farmers transitioned to high-yielding practices based on the regional survey, but a number of indicators decreased in performance when evaluating management packages to further increase production levels for high-yielding farmers in the on-farm trials. In contrast to the environmental tradeoffs noted above, BMPP significantly reduced YSACR by 6.6 to 18.3% in the first stage trials and 11.2% in the second stage trials. These results highlight the possibility of further increasing yield without increasing the risk of freshwater agrochemical contamination per unit production. Here we used USEtox to estimate the environmental impacts of agrochemicals, but similar conclusions have been reached using different criteria such as treatment frequency index (TFI)^57^.

Holistic approaches accounting for synergies and tradeoffs (e.g. agrochemical contamination risk vs. NUE in this study) within different intensification levels should help prevent bias in promoting one indicator over another, which also benefits subsequent policing-making^58^. Regarding opportunities for SI in average vs. high-yielding intensification levels, shifting to more intensive management to further increase productivity at high baseline yield levels was less likely to achieve the dual goal of SI, suggesting investments focusing on average-yielding farmers while maintaining the production levels of high-yielding farmers might be a promising strategy for SI planning in this context. However, to strengthen such efforts in Uruguay and other regions, indicators reflecting other dimensions of environmental and socioeconomic sustainability are clearly needed. For example, the present study has limitations because we focused on input-related impacts which does not account for other contributing factors and dimensions. Future work should also include the effects of changes in management on other SI components. For a comprehensive list of potential indicators, the reader is referred to other recent publications^40,41,59,60^.

### Remarks on relevant but missing aspects

There are several important and relevant aspects we did not investigate in this study. This study addressed the tradeoffs between productivity and environmental sustainability from a co-designed research approach between farmers and agronomists, but our experiments did not reflect all possible production conditions and we did not evaluate interannual yield variability. Although the proposed BMPP increased yield in 2 consecutive trials, it is uncertain how this practice would perform under variable climate conditions in the future. Given that yield stability has been reported as an important characteristic for farmers in other cereal systems^59^, future research across a wider range of environments and seasons should be conducted to assess yield stability performance.

Additionally, water usage-related metrics were not investigated in this study but should be included in future studies on sustainable rice production in Uruguay. Zampieri *et al*.^61^ reported the recent changes in management practices (postponed flooding) might cause water-related stress on long-term sustainability, despite water availability has not yet been a limiting factor of production. As Italy shares multiple common characteristics with Uruguay rice systems (lack of water limitation, dry-seeding establishment and industrialized production), and recent studies suggest there is potential to improve water productivity without sacrificing yield in Uruguay^62^, the water-related outcomes of changes in crop management and future yield improvements requires more attention to ensure long-term sustainability.

### Pursuing sustainable intensification in high-yielding cereal systems: a challenging yet unattended task

Closing yield gaps is considered as important means of enhancing food security and agricultural sustainability^16,63^. In most SI research, relationships between yield and environmental footprint are not addressed with the awareness of how much yield gap remains in the system. Carracelas *et al*.^64^ reported the rice yield potential in two regions of eastern Uruguay are 13.7 Mg ha^−1^ and 14.7 Mg ha^−1^, making the average attainable yield 11.4 Mg ha^−1^. Thus, the exploitable yield gap for average-yielding farmers, the HYFP treatment, and the BMPP treatment in the second stage trials can be estimated at 3.8, 2.4 and 1 Mg ha^−1^, respectively. While it is often assumed that closing yield gaps will increase resource use efficiencies, Noordwijk *et al*.^65^ reported that the relationship between yield gaps and resource use efficiency gaps is uncertain with mixed empirical evidence.

Within the context of Uruguay, we found that opportunities for simultaneously increasing yield and environmental sustainability differed depending on the baseline for comparison at the two levels of intensification (i.e. average-yielding vs. high-yielding farmers). For average-yielding farmers, adopting HYFP may improve both yield and environmental efficiencies, largely due to yields increasing faster than corresponding changes in environmental impact. In contrast, tradeoffs were found when evaluating options to increase productivity for high-yielding farmers using BMPP, specifically related negative impacts on carbon, energy, and N footprints. Assuming that yield increases become harder to achieve as cereal systems move closer to the yield ceiling, these results imply that making progress towards the dual goals of SI is inherently more challenging in high-yielding systems. This distinction is not typically considered by researchers and policy-makers working to promote SI at a global scale.

Currently, most SI research is conducted in less developed regions^42^, where yield gaps are often large. In these contexts, the biological yield ceiling is less relevant because large yield gains can occur with relatively modest changes in crop management. In contrast, Grassini *et al*.^16^ reported that around 31% of global major cereals (rice, wheat and maize) are produced in regions where yield growth has stagnated. In these regions, economic investments continue to be made to further improve productivity (e.g. Zhang *et al*.^66^), suggesting that more resources are dedicated to addressing the production side of SI with fewer resources dedicated to addressing environmental dimensions. Yet our findings indicate that it is precisely systems with small yield gaps that may experience greater tradeoffs in resource use efficiencies and environmental indicators when trying to further raise yields compared to a high baseline. In such cases, some level of prioritization between sustainability and production goals will likely be necessary to make the best use of limited resources, particularly considering that overall investment in public agricultural research and improving yield is diminishing^16,67^. Further work quantifying potential gains in high-yielding vs. low-yielding systems could inform such investments, helping determine whether increased emphasis on environmental dimensions would be more cost-effective in high-yielding systems.

The long-term vision of SI is re-designing current agroecosystems^12^. Despite a number of examples having shown that SI in intensified systems is achievable^68,69^, changing the current paradigm has many challenges. In developed countries, barriers for implementing novel farming technologies have been identified and described as a “lock-in” condition^70,71^. This is in part due to existing socioeconomic relationships and interactions between consumers, farmers, and service providers. In contrast, a recent study in a highly-productive area of India (e.g. Kumar *et al*.^69^) concluded that simultaneously increasing productivity, profitability and sustainability is possible by incorporating novel crops into the system, yet shifting the value chain and public procurement are still necessary steps to support such changes. Thus, transformational changes are clearly necessary to achieve SI, but at the same time these changes will require long-term effort and resources to implement. This highlights an important opportunity for short-term actions that have the capability to accelerate progress towards SI in cropping systems nearing yield potential.

From a feasibility standpoint, the approach used in this study represents an economically efficient research framework for quickly enhancing SI activities in mainstream cereal production systems. Although yield in Uruguay is currently prioritized against other environmental goals to maintain competitiveness in the global marketplace, this study illustrates the potential of building on an existing research and extension program as a starting point for balancing productivity and sustainability outcomes. Such an approach would be particularly useful in mainstream agricultural research programs where the emphasis is on productivity and conversations between agronomists and others interested in environmental sustainability remain limited.

## Conclusion

Pathways for SI in high-yielding Uruguayan rice production systems were explored in this study following two distinct approaches. First, current yield levels and management practices for average farmers were compared to high-yielding farmers to evaluate potential improvements in production as well as corresponding impacts on sustainability indicators based on farmer surveys and crop management records at the regional-scale. Second, on-farm field experiments were conducted in two stages to evaluate options for further increasing yield relative to that currently achieved by high-yielding farmers. In general, field study results showed there is little room to boost production of the top-yielding farmers from an environmental perspective, at least considering the management practices tested here, as no treatment simultaneously improved yield and the full suite of sustainability indicators evaluated in this study compared to HYFP. In contrast, survey results indicate there is room to continue improving yields of the average farmers, with high-yielding farmers having higher NUE and net energy yield but lower carbon and agrochemical footprint per kg of rice yield. Considering the global challenge of further increasing yields in cereal production systems nearing the yield ceiling, this study provides an important example of transforming a current yield improvement program into a platform for evaluating SI goals with relatively low cost and effort. While there are limitations to consider, we suggest that one opportunity for more broadly advancing SI research in mainstream cereal production systems is by including an ex-post analysis of environmental indicators in field research trials focused on increasing yields.

## Supplementary information

Supplementary information

Supplementary information 2

## Data Availability

The detailed data and unaggregated results of the first and second stage field trials are included in Supplementary table S5.
