# Supplementary material for: Towards actionable research frameworks for sustainable intensification in high-yielding rice systems
Source: Sci Rep. 2020 Jun 19;10:9975. doi: 10.1038/s41598-020-63251-w (PMC7305151; doi:10.1038/s41598-020-63251-w)
Supplement: Supplementary file 1 — Supplementary information [file 41598_2020_63251_MOESM1_ESM.docx]

**Towards actionable research frameworks for sustainable intensification in high-yielding rice systems**

**Meng-Chun Tseng^1*^, Álvaro Roel^2^, Enrique Deambrosi^2^, José A. Terra^2^, Gonzalo Zorrilla^2^, Sara Riccetto^1^, Cameron M. Pittelkow^1,3^**

^1^Department of Crop Sciences, University of Illinois, Urbana, Illinois 61801 USA

^2^National Institute of Agricultural Research (INIA), Treinta y Tres, Uruguay

^3^Department of Plant Sciences, University of California, Davis, CA 95616 USA

*Corresponding author: mctseng2@illinois.edu

**List of supplementary information**

As some of the supplementary table and data contain complex multiple-year location trials information, the authors keep those data in excel file format. All the supplementary information was compressed into a single zip file. This list is the guide to identify the individual file under the zip folder for desired table, figure and data.

**Figure S1a - Detailed locations of first stage trial**

File: Figure S1 - Detailed trial locations.pdf

**Figure S1b – Detailed locations of second stage trial**

File: Figure S1 - Detailed trial locations.pdf

**Table S1**

**Farm-level Yield of surveyed high-yielding rice farmers in the three main production regions in Eastern Uruguay**

File: Table S1 S2.docx

**Table S2**

**Consensus management practices for high-yielding rice farmers based on surveys administered in the three main production regions in Eastern Uruguay and a joint meeting with representatives from INIA, the rice farmers association (ACA), and commercial rice mills**

File: Table S1 S2.docx

**Table S3**

**Detailed treatment information of the first-stage trials**

File: Table S3 S4-FieldTrialsTreatments.xlsx

**Table S4**

**Detailed treatment information of the second-stage trials**

File: Table S3 S4-FieldTrialsTreatments.xlsx

**Table S5a**

**Unaggregated results of first and second stage field trials**

File: Table S5a-Unaggregated results of first and second stage field trials.xlsx

**Table S5b**

**Region level results of first stage trials**

File: Table S5b-Region_level_results_first_stage_trials.xlsx

**Table S5c**

**Region level results of second stage trials**

File: Table S5c-Region_level_results_second_stage_trials.xlsx

**File S1**

**Agrochemical contamination risk of applied pesticides**

File: File S1- Agrochemical contamination risk of applied pesticides.xlsx
