# Supplementary material for: Towards actionable research frameworks for sustainable intensification in high-yielding rice systems
Source: Sci Rep. 2020 Jun 19;10:9975. doi: 10.1038/s41598-020-63251-w (PMC7305151; doi:10.1038/s41598-020-63251-w)
Supplement: Supplementary file 2 — Supplementary information 2 [file 41598_2020_63251_MOESM2_ESM.zip › Table S1 S2.docx]

**Towards sustainable intensification in high-yielding cereal systems**

**Meng-Chun Tseng^1*^, Álvaro Roel^2^, Enrique Deambrosi^2^, José A. Terra^2^, Gonzalo Zorrilla^2^, Sara Riccetto^1^, Cameron M. Pittelkow^1^**

^1^Department of Crop Sciences, University of Illinois, Urbana, Illinois 61801 USA

^2^National Institute of Agricultural Research (INIA), Treinta y Tres, Uruguay

*Corresponding author: mctseng2@illinois.edu

**Supplementary Table S1**. Farm-level Yield of surveyed high-yielding rice farmers in the three main production regions in Eastern Uruguay

| Region | Farmer ID | Season | | |
| --- | --- | --- | --- | --- |
|  |  | 2010-2011 | 2011-2012 | 2012-2013 |
| **Treinta y Tres** | 19 | 9522.5 | 6513 | 9787 |
|  | 1 | 9750 | 8950 | 9150 |
|  | 2 | 9100 | 8700 | 9250 |
|  | 28 | 8981.5 | 7052.5 | 8623 |
|  | 24 | 9812.5 | 8229 | 9061 |
|  | 14 | 9512 | 8805 | 8773 |
|  | 21 | 10392.5 | 8989.5 | 9159 |
|  | 22 | 9622 | 8262.5 | 9134.5 |
|  | 5 | 10000 | 8150 | 8600 |
|  | 23 | 9718 | 7450 | 8816 |
|  | 4 | 10600 | 9150 | 9100 |
|  | 20 | 8448 | 7837 | 8835.5 |
|  | 17 | 10249 | 8680 | 9099.5 |
|  | 25 | 9745.5 | 9088 | 9113.5 |
|  | 26 | 9731.5 | 8799 | 8031 |
|  | **Average** | **9679** | **8310.36667** | **8968.86667** |
| Cebollatí | 36 | 9100 | 8850 | 8500 |
|  | 32 | 8650 | 8700 | 8600 |
|  | 6 | 9550 | 8782 | 9705.5 |
|  | 33 | 8750 | 7500 | 9000 |
|  | 7 | 10069 | 9120 | 8626 |
|  | 13 | 8700 | 8834.5 | 8040 |
|  | 15 | 10174.5 | 9381 | 9788 |
|  | 16 | 10247.5 | 10416 | 9752 |
|  | 11 | 9767 | 8576.5 | 10051 |
|  | 34 | 10300 | 8600 | 9150 |
|  | 37 | 8500 | 8150 | 8400 |
|  | 38 | 8500 | 8650 | 9600 |
|  | 39 | 9300 | 8300 | 8250 |
|  | 18 | 9893 | 7906.5 | 8322.5 |
|  | **Average** | **9392.92857** | **8697.60714** | **8984.64286** |
| India Muerta | 29 | 8650 | 8550 | 8500 |
|  | 30 | 8200 | 8050 | 8550 |
|  | 10 | 9421.5 | 8855.5 | 9229.5 |
|  | 31 | 9050 | 8850 | 8500 |
|  | 8 | 9434.5 | 9392 | 9749 |
|  | 9 | 9382 | 8394.5 | 10023 |
|  | 12 | 9644.5 | 8380 | 9483 |
|  | 35 | 9050 | 8000 | 8650 |
|  | 3 | 10600 | 8800 | 10000 |
|  | 40 | 9550 | 8950 | 8850 |
|  | **Average** | **9298.25** | **8622.2** | **9153.45** |

**Supplementary Table S2**. Consensus management practices for high-yielding rice farmers based on surveys administered in the three main production regions in Eastern Uruguay and a joint meeting with representatives from INIA, the rice farmers association (ACA), and commercial rice mills.

| **Group** | **Trienta y Tres** | | **Cebollati** | | **India Muerta** |
| --- | --- | --- | --- | --- | --- |
| Farm Size (ha) | 300-500 | | 100-300 | | 100-300 |
| Pre-plant glyphosate application | Spring | | Spring | | Spring and previous fall |
| Variety | EP144 | | EP144 | | INIA Tacuari |
| Planting date initiation | Before 10 October | | Before 15 October | | Before 20 October |
| Summer tillage (coming out of pasture the previous year) | Yes | | Yes | | Yes |
| Previous land use | 1-2 years not improved pastures | | 3-4 years not improved pastures | | 3 or more years improved pastures |
| Tillage | Reduced tillage or no tillage | | Conventional | | Conventional |
| Seeding rate (kg ha^-1^) | 130 | | 160 | | 170 |
| Seed treatment | Fungicide + insecticide | | Fungicide + insecticide | | Fungicide + insecticide |
| Basal fertilizer application | Broadcasted before planting | | Applied with planter at seeding | | Applied with planter at seeding |
| Basel fertilizer rate (kg ha^-1^) | N:10-15  P_2_O_5_: 50-60  K_2_O: 20-30 | | N:19.8  P_2_O_5_: 506 | | N:19.8  P_2_O_5_: 50.6 |
| 1^st^ urea top dress | 75 kg ha^-1^ at tillering before flooding | | 60 kg ha^-1^ at tillering before flooding | | 60 kg ha^-1^ at tillering before flooding |
| 2^nd^ urea top dress | 50 kg ha^-1^ at panicle initiation | | 50 kg ha^-1^ at panicle initiation | | 50 kg ha^-1^ at panicle initiation |
| Pre-emergence glyphosate + clomazone | Yes | | No | | No |
| Herbicide (post-emergence) | Penoxsulam | | Penoxsulam | | Clomazone + Quinclorac + Propanil |
| Fungicide application method | 1^st^ application at heading + 2^nd^ application at 15-20 days after heading (if blast symptoms are found) | | | | |
| Fungicide | tebuconazole + trifloxystrobin | | | | |
| Vertical interval among levees (cm) and relative levee size | 3-6 (short) | 3-6 (medium) | | 3-6 (short) | |
| Timing of building levees | Before seeding | Right at seeding | | Right at seeding | |
| Days to flood after emergence | 25 | 30-35 | | 30 | |
| Drain water for harvest | Yes | Yes | | Yes | |
