# Supplementary figures and images for: Towards actionable research frameworks for sustainable intensification in high-yielding rice systems
Source: Sci Rep. 2020 Jun 19;10:9975. doi: 10.1038/s41598-020-63251-w (PMC7305151; doi:10.1038/s41598-020-63251-w)

a

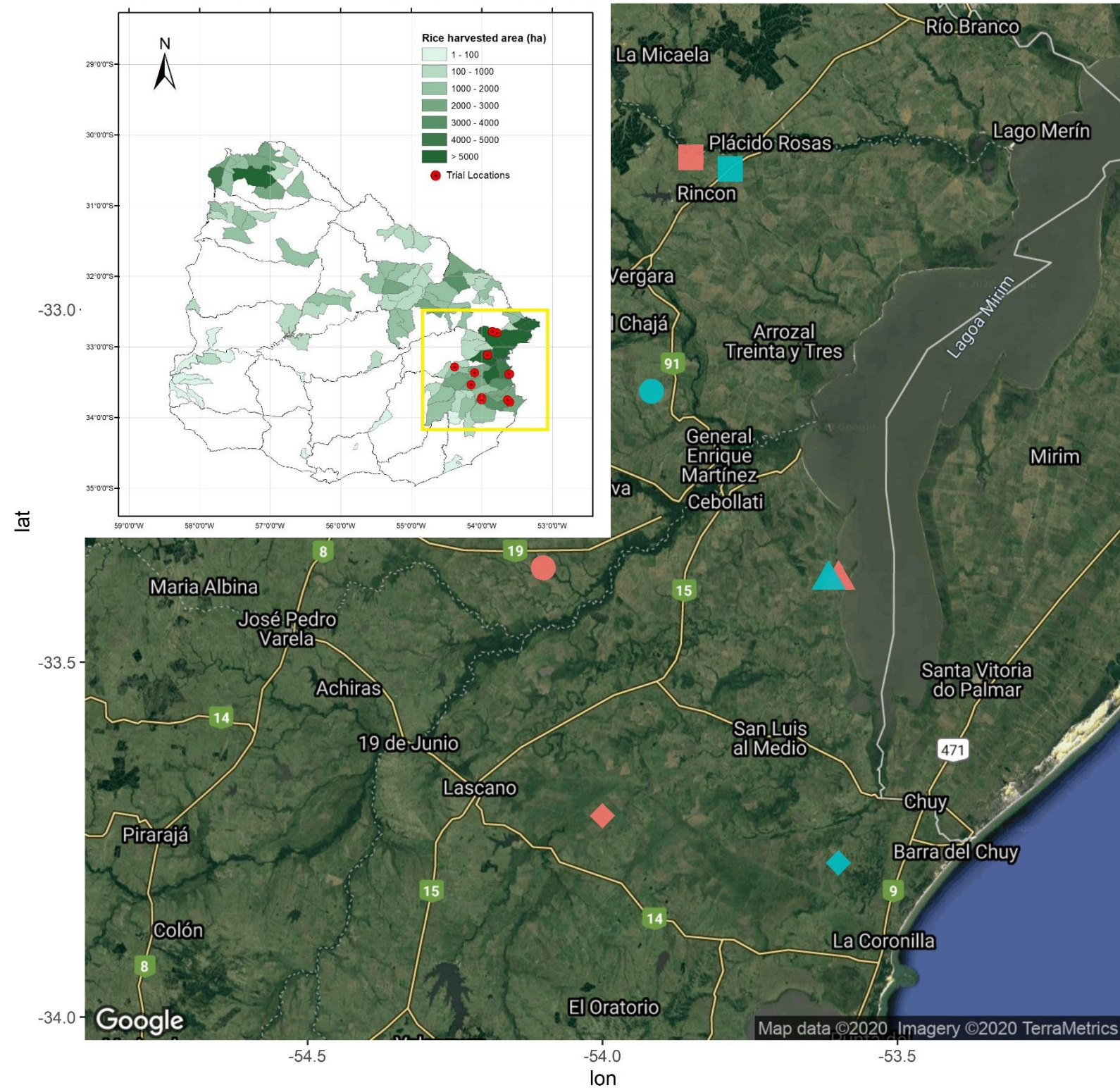

b

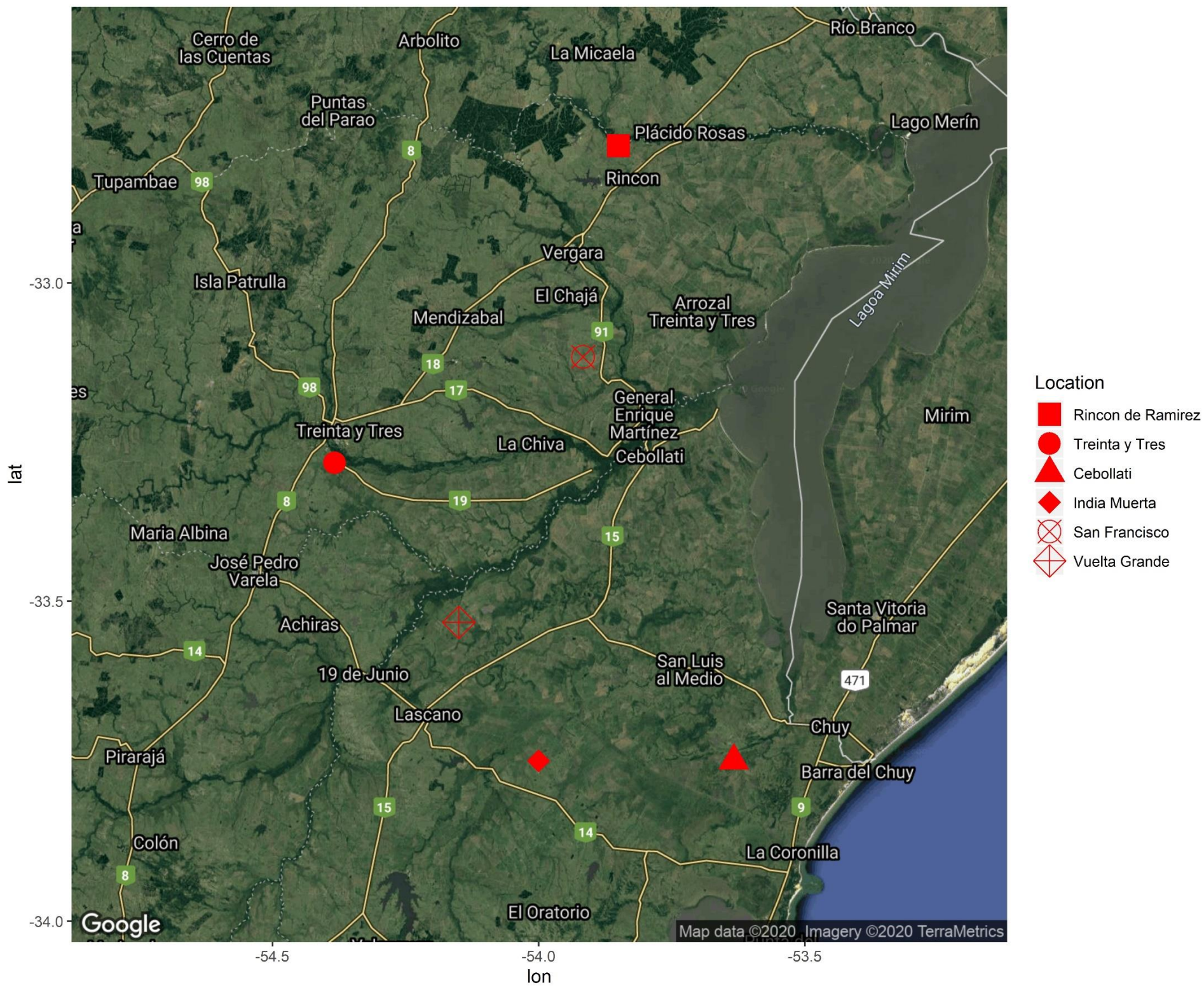

Supplement: Supplementary file 2 — Supplementary information 2 [file 41598_2020_63251_MOESM2_ESM.zip › Figure S1 - Detailed trial locations.pdf]
